# Supplementary figures and images for: FAM81A is a postsynaptic protein that regulates the condensation of postsynaptic proteins via liquid–liquid phase separation
Source: PLoS Biol. 2024 Mar 7;22(3):e3002006. doi: 10.1371/journal.pbio.3002006 (PMC10919877; doi:10.1371/journal.pbio.3002006)

Figure S1

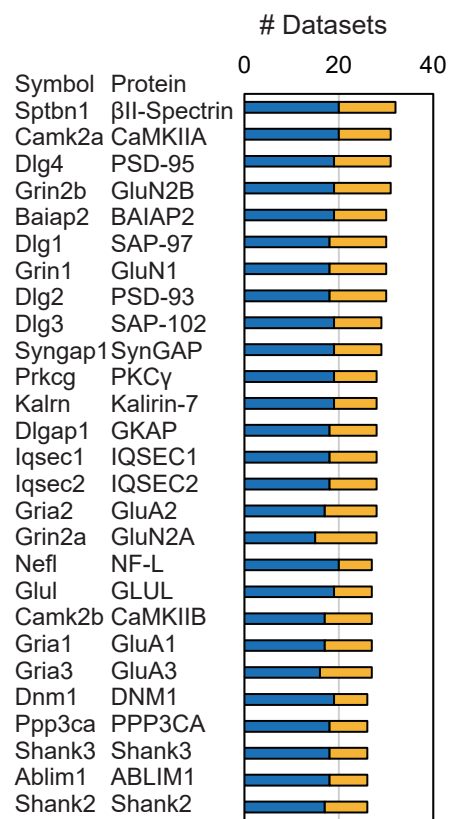

Supplement: S1 Fig — List of the top 27 proteins that show the highest number of datasets. Blue and yellow bars indicate the dataset number of unbiased and candidate-based approaches. Proteins detected in at least 26 datasets are listed. (PDF) [file pbio.3002006.s001.pdf]

Figure S2

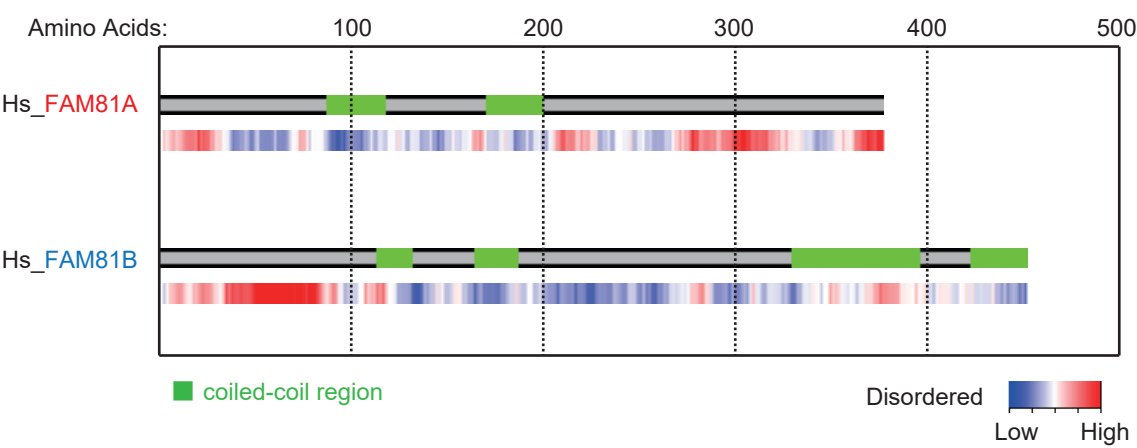

Supplement: S2 Fig — Coiled-coil regions detected by SMART and disordered regions predicted by IUPred2A. (PDF) [file pbio.3002006.s002.pdf]

Figure S3

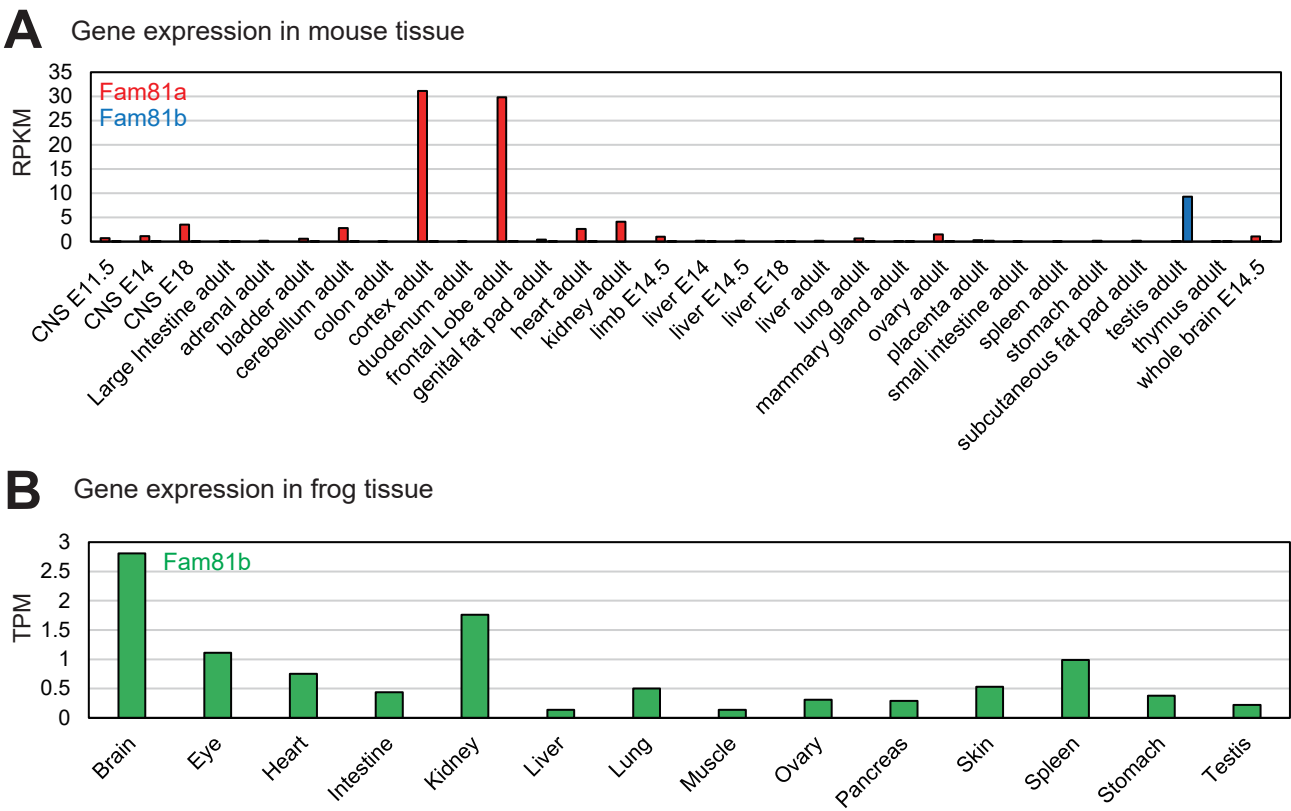

Supplement: S3 Fig — (A and B) Gene expression pattern of FAM81A and FAM81B in mouse (A) and frog (B) tissue. Mouse data and frog data were obtained from NCBI Gene and Xenbase, respectively. RPKM: Reads per Kilobase of exon per Million mapped reads; TPM: Transcripts per Kilobase Million. (PDF) [file pbio.3002006.s003.pdf]

Figure S4

**A**

IHC: FAM81A (Ab2)

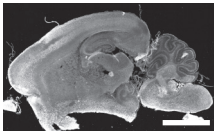

**B**

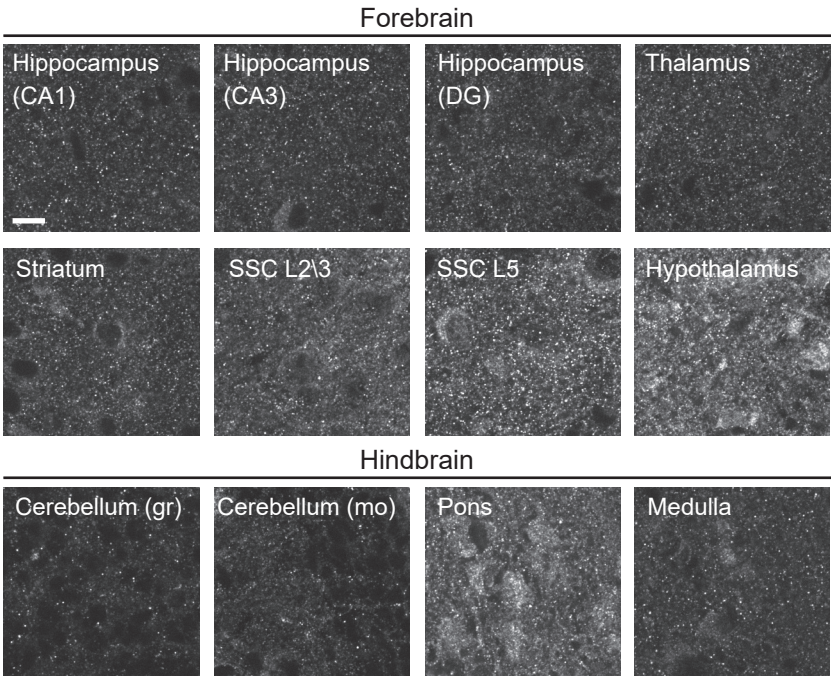

Supplement: S4 Fig — (A and B) Distribution of FAM81A in brain. Immunohistochemistry of FAM81A was performed on sagittal section of postnatal day 7 mouse brain. Scale bar: 2 mm (A) and 10 μm (B). (PDF) [file pbio.3002006.s004.pdf]

Figure S5

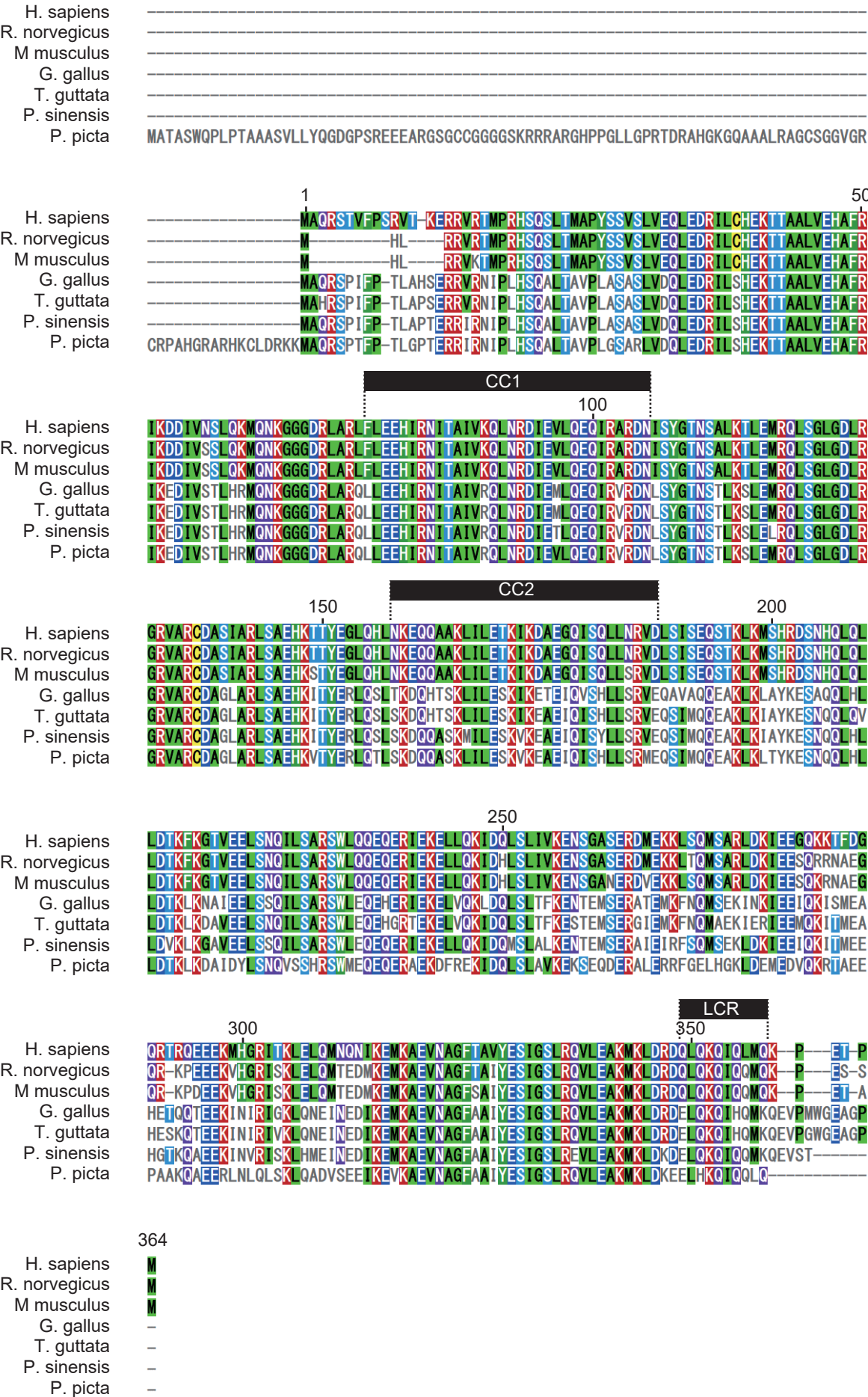

Supplement: S5 Fig — The sequence of FAM81A homologs of indicated species are aligned using Kalign and visualized using MView. The numbers on the sequences indicate the amino acid number of mouse FAM81A. Residues identical to human FAM81A are highlighted. The colors of the characters represent the classification of amino acids: hydrophobic (light green), large hydrophobic (dark green), positive (red), small alcohol (light blue), and polar (purple). Mammals: human (Homo sapiens), rat (Rattus norvegicus), and mouse (Mus musculus). Birds: chicken (Gallus gallus) and zebra finch (Taeniopygia guttata). Reptiles: soft-shelled turtle (Pelodiscus sinensis) and gecko (Paroedura picta). The coiled-coil domains (CC1 and CC2) and low complexity region (LCR) of mouse FAM81A are labeled with a black bar. (PDF) [file pbio.3002006.s005.pdf]

Figure S6

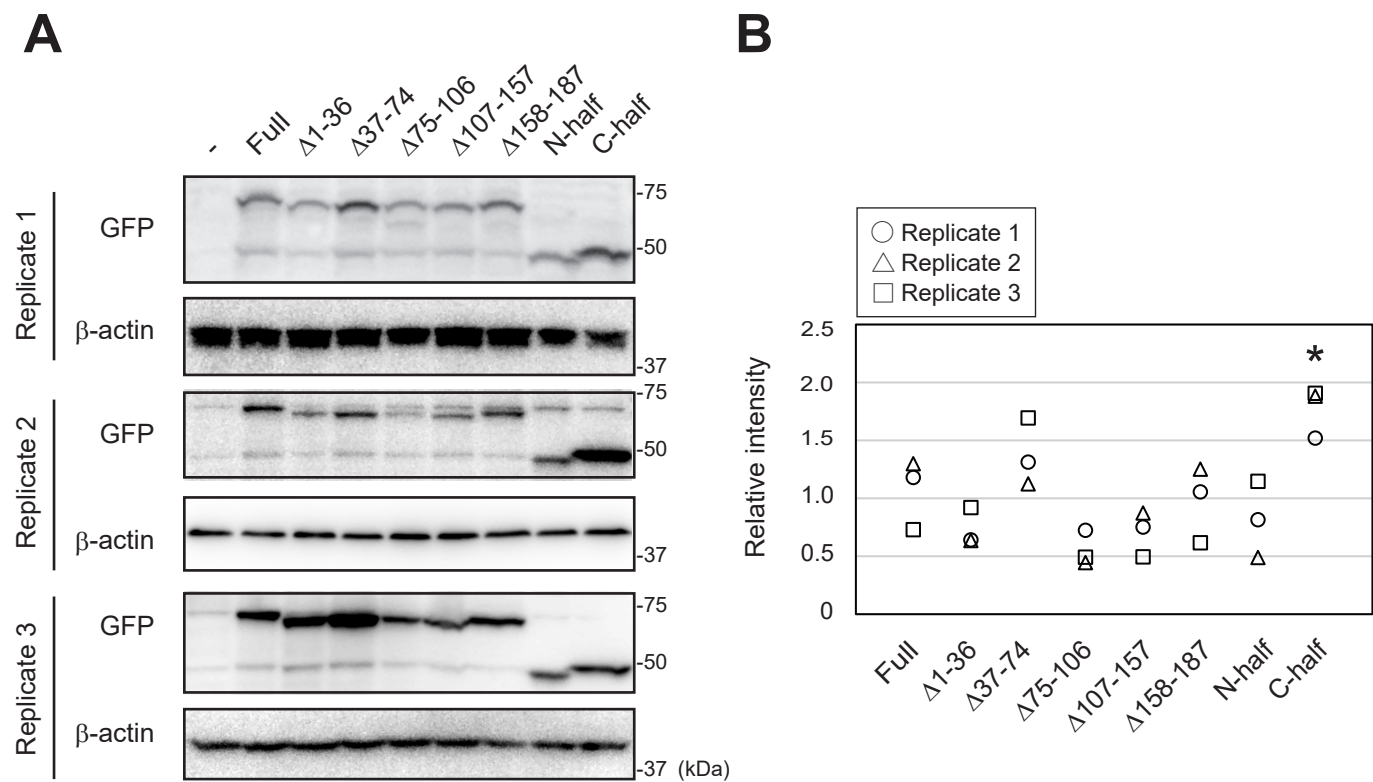

Supplement: S6 Fig — (A) HEK293T cells were transfected with FAM81A-GFP or its mutant 24 h later, cells were lysed and protein concentration was quantified, and 30 μg protein was subjected to immunoblotting using anti-GFP or anti-β-actin antibodies. (B) Relative band intensity of panel A data quantified using ImageJ. *P < 0.05, unpaired Student’s t test. (PDF) [file pbio.3002006.s006.pdf]

Figure S7

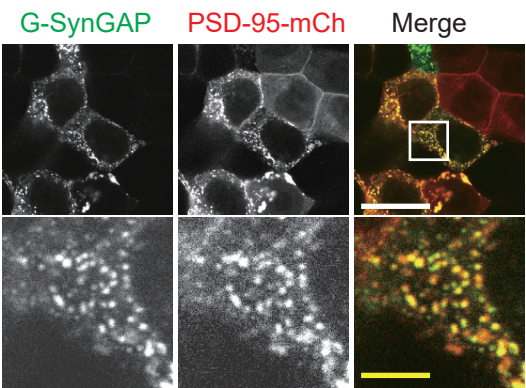

Supplement: S7 Fig — HEK293T cells were transfected with indicated plasmids, and 24 h later, cells were fixed and observed with confocal microscopy. Scale bars: 20 μm (white) or 4 μm (yellow). (PDF) [file pbio.3002006.s007.pdf]

Figure S8

**A**

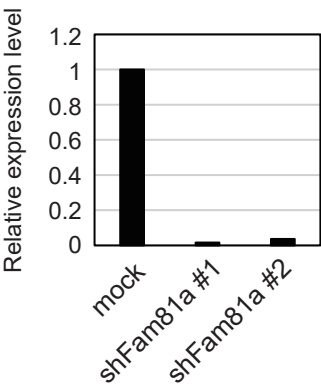

**B**

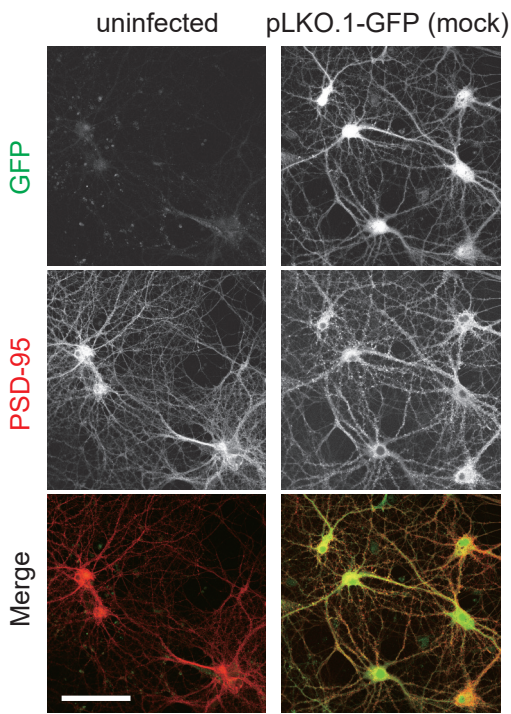

Supplement: S8 Fig — (A) Primary cultured mouse cortical neurons were infected with lentivirus encoding FAM81A shRNAs at DIV4. At DIV16, neurons were harvested to extract mRNA. After the preparation of cDNA, real-time PCR was performed. The relative expression level is described. (B) Primary cultured mouse hippocampal neurons were infected with lentivirus of pLKO.1-GFP mock plasmid at DIV14. At DIV21, the neurons were fixed and subjected to immunocytochemistry using an anti-PSD-95 antibody. Scale bars: 100 μm. (PDF) [file pbio.3002006.s008.pdf]
