# Supplementary material for: FAM81A is a postsynaptic protein that regulates the condensation of postsynaptic proteins via liquid–liquid phase separation
Source: PLoS Biol. 2024 Mar 7;22(3):e3002006. doi: 10.1371/journal.pbio.3002006 (PMC10919877; doi:10.1371/journal.pbio.3002006)
Supplement: S1 Raw Images — (PDF) [file pbio.3002006.s015.pdf]

Figure 3A

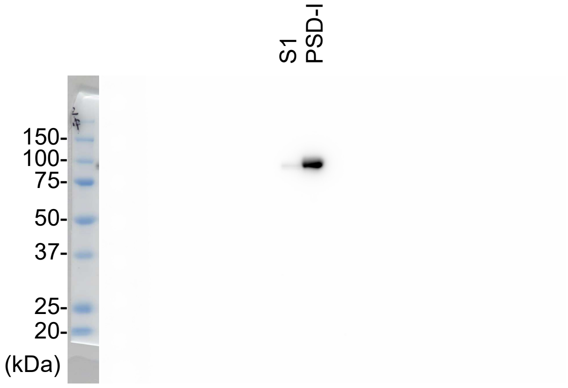

PSD-95 (Abcam, ab18258)

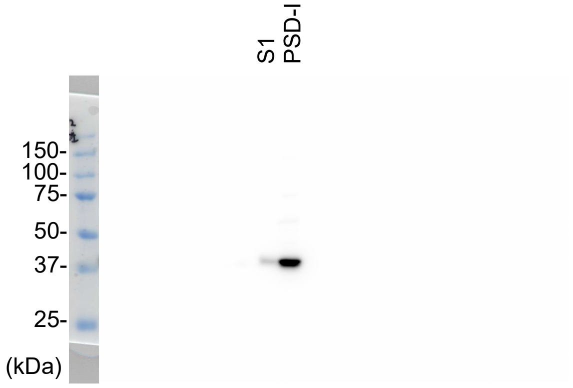

FAM81A (Ab1) (Novus, NBP2-33295)

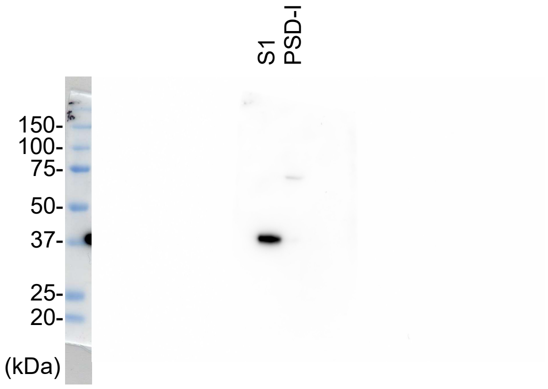

Synaptophysin (Cell Signaling, #4329)

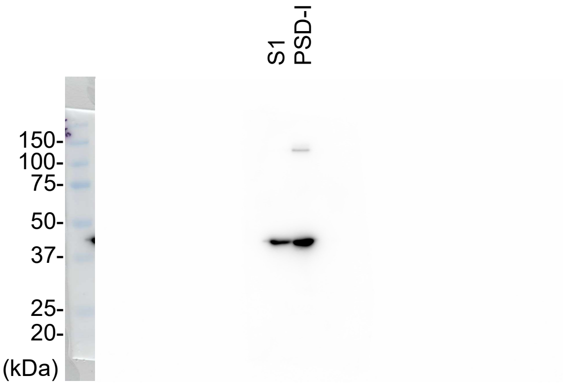

β-actin (SIGMA, A1978)

Figure 3B

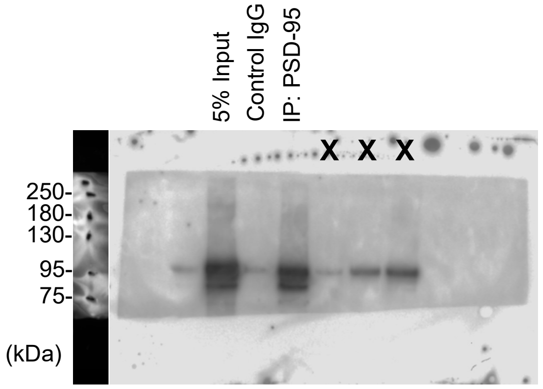

PSD-95 (NeuroMab, 75-028)

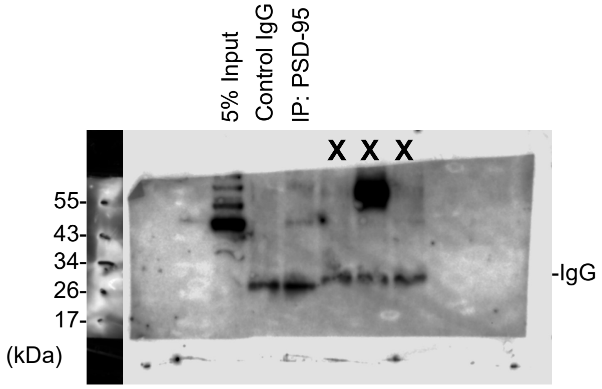

FAM81A (Ab2) (MERCK, HPA065797)

Figure 6C

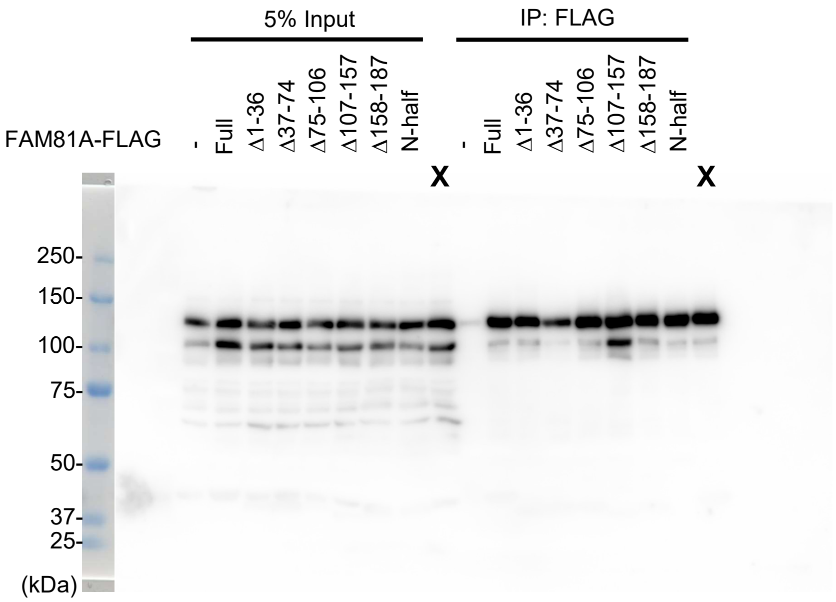

GFP (Thermo Fisher Scientific, A-6455)

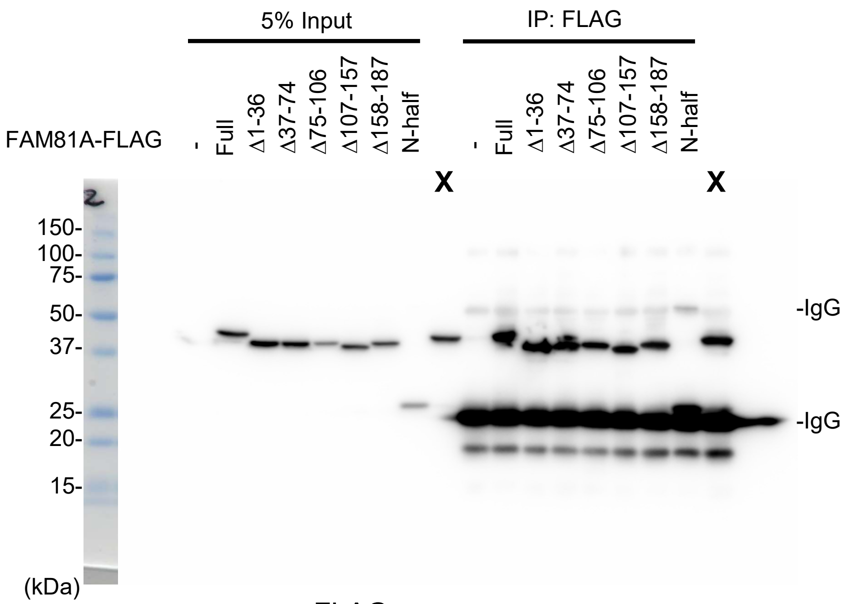

FLAG (SIGMA, F3165)

Figure 7F

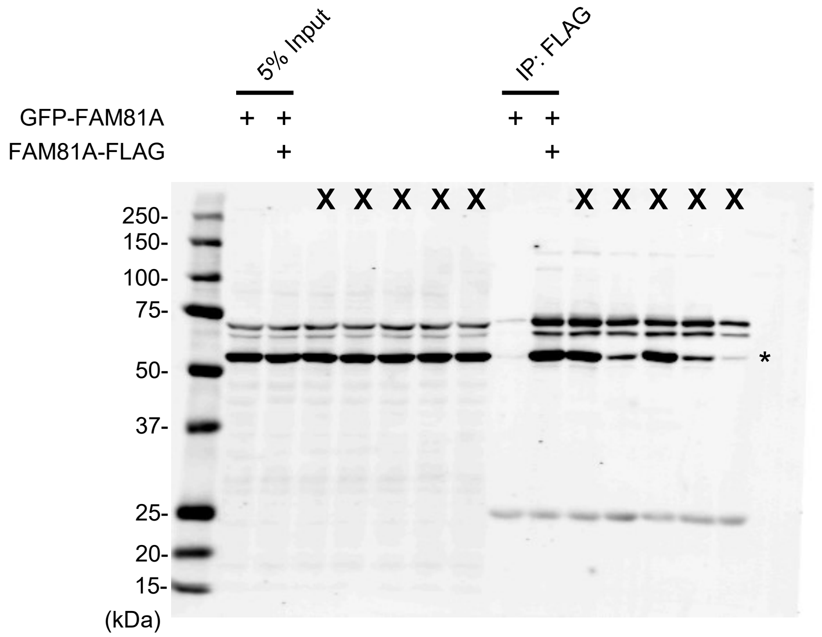

GFP (Thermo Fisher Scientific, A-6455)

* We think this band is a partial degradation product of GFP-FAM81A

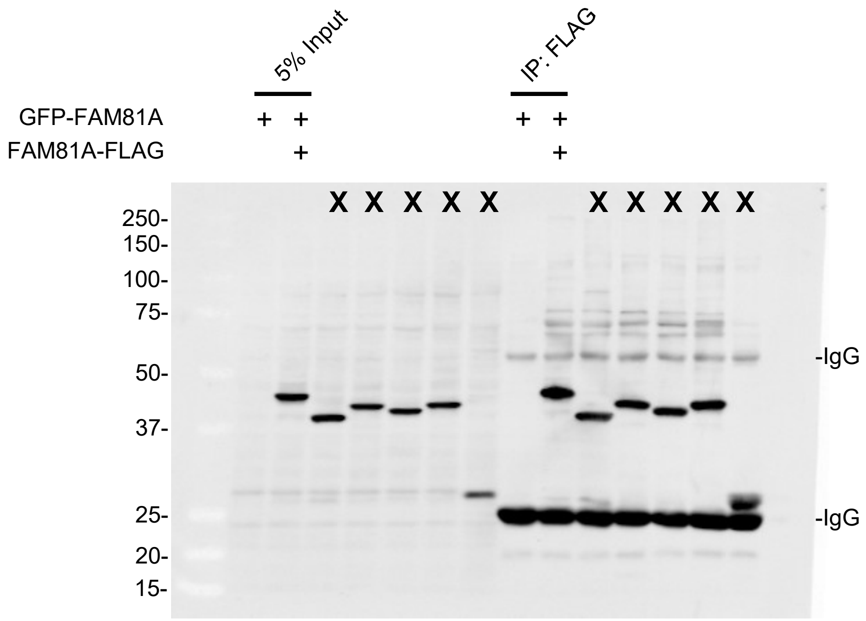

FLAG (SIGMA, F3165)

Figure 7G

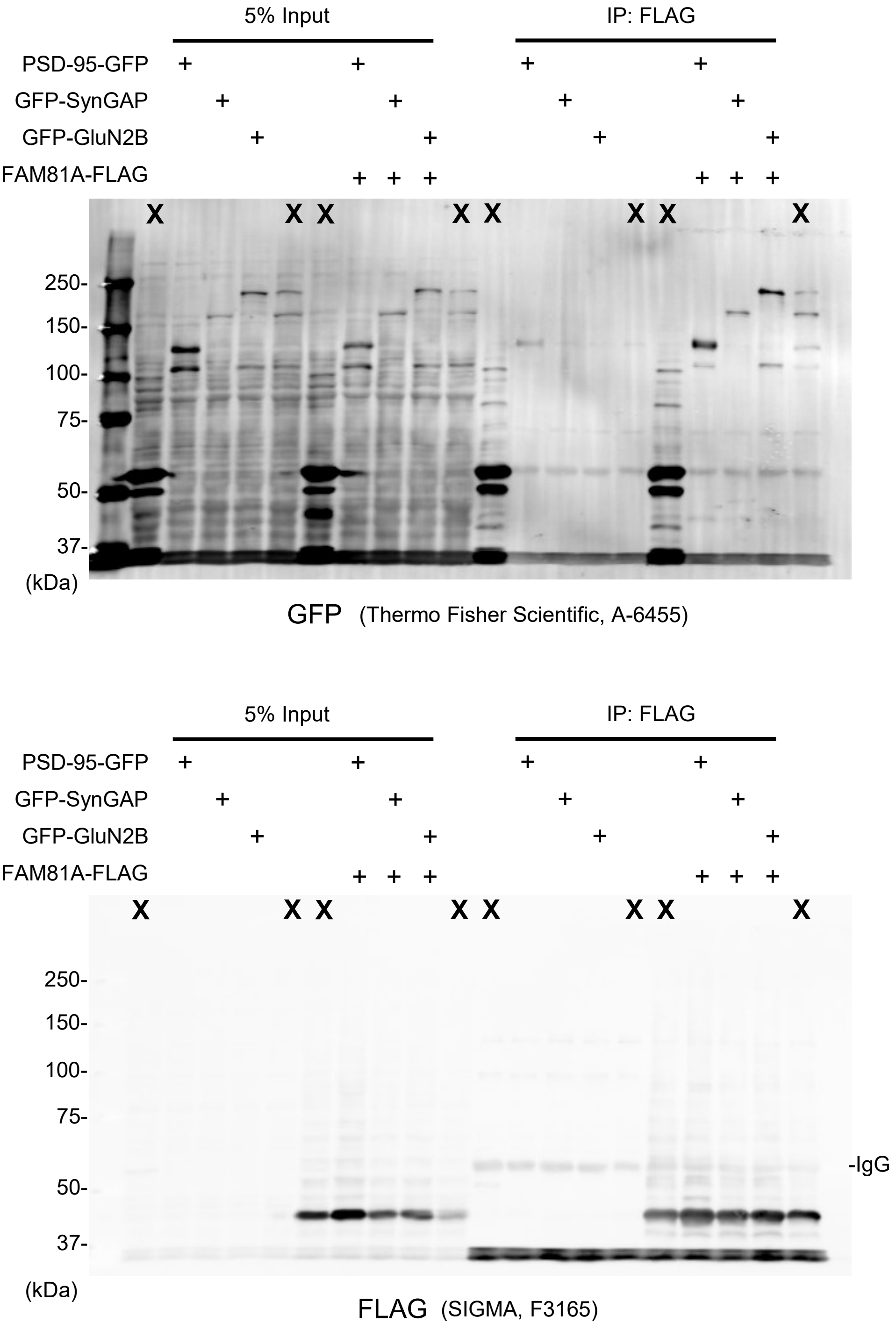

5% Input
IP: FLAG
PSD-95-GFP
GFP-SynGAP
GFP-GluN2B
FAM81A-FLAG
250-
150-
100-
75-
50-
37-
(kDa)
GFP (Thermo Fisher Scientific, A-6455)
5% Input
IP: FLAG
PSD-95-GFP
GFP-SynGAP
GFP-GluN2B
FAM81A-FLAG
250-
150-
100-
75-
50-
37-
(kDa)
-IgG
FLAG (SIGMA, F3165)

Figure S6A

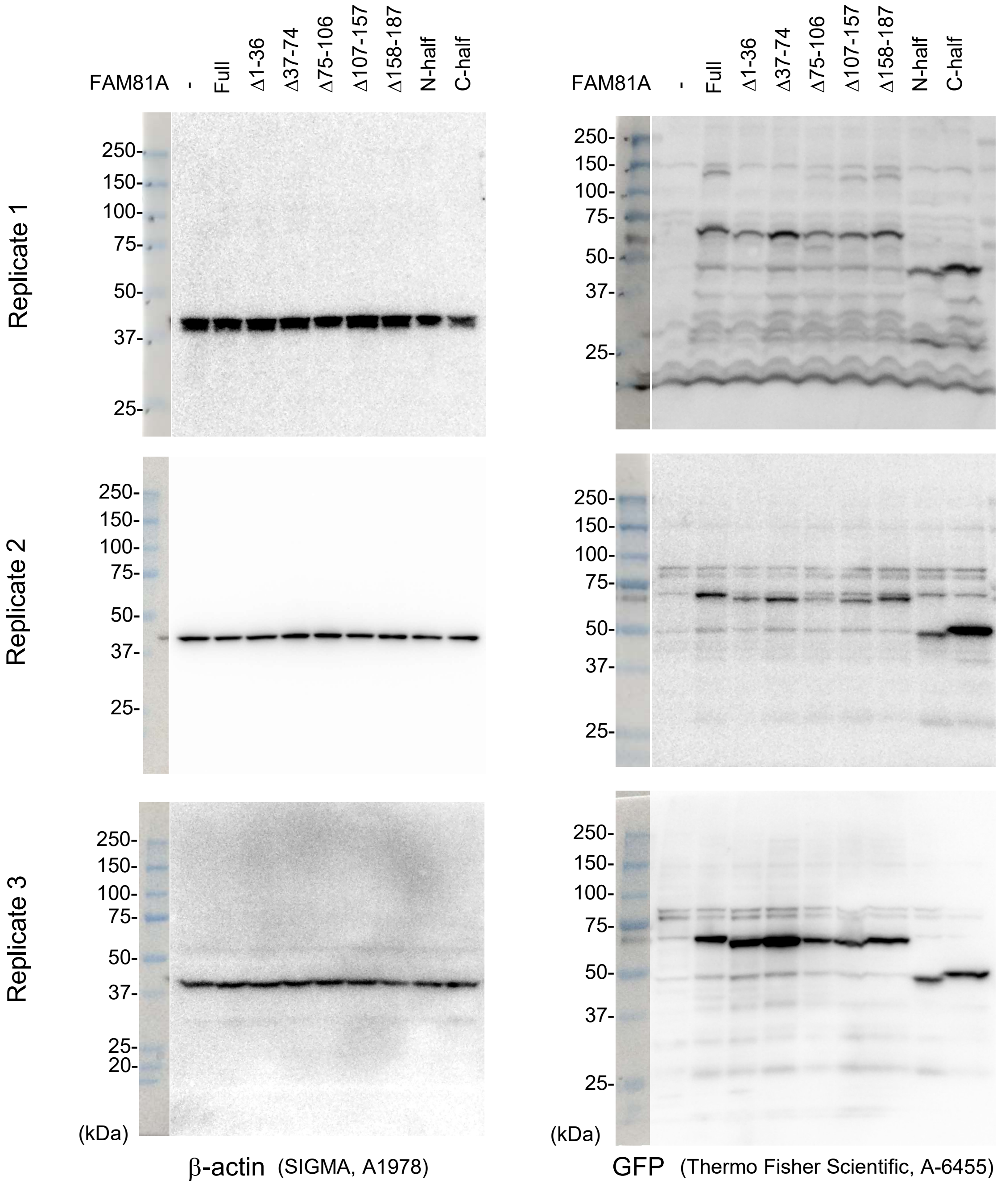
FAM81A
-
Full
Δ1-36
Δ37-74
Δ75-106
Δ107-157
Δ158-187
N-half
C-half
Replicate 1
Replicate 2
Replicate 3
250-
150-
100-
75-
50-
37-
25-
20-
(kDa)
β-actin (SIGMA, A1978)
GFP (Thermo Fisher Scientific, A-6455)
