# Supplementary material for: FAM81A is a postsynaptic protein that regulates the condensation of postsynaptic proteins via liquid–liquid phase separation
Source: PLoS Biol. 2024 Mar 7;22(3):e3002006. doi: 10.1371/journal.pbio.3002006 (PMC10919877; doi:10.1371/journal.pbio.3002006)
Supplement: Attachment — Submitted filename: Kaizuka_FAM81A_Rebuttal_231024.docx [file pbio.3002006.s016.docx]

# Point-by-point response to the Editor and reviewers

The editor’s and reviewer’s comments are reproduced in italics, with our responses in normal text.

**Editor, point 1:**

*Dear Dr Takumi,*

*Thank you for your patience while your manuscript "DRACC1, a major postsynaptic protein, regulates the condensation of postsynaptic proteins via liquid-liquid phase separation" was peer-reviewed at PLOS Biology. Your manuscript has been evaluated by the PLOS Biology editors, an Academic Editor with relevant expertise, and by several independent reviewers.*

*As you will see in the reviewer reports, which can be found at the end of this email, although the reviewers find the work potentially interesting, they have also raised a substantial number of important concerns. We think that, in order to offer the strength of advance that we would require for publication at PLOS Biology, these concerns would need to be thoroughly addressed, with new data and analyses where appropriate.*

We thank all the editor and reviewers for the critical reading of our manuscript. We are pleased that reviewers found our study interesting. To address the concerns, we performed multiple experiments, analyses, and rewriting. We believe that the revised manuscript now meets the requirements for publication in PLOS Biology.

**Editor, point 2:**

*After discussion with the Academic Editor we think the study would need to be expanded to test the expression of Fam81A, in vivo, as Reviewer 1 has suggested. Additionally, we feel that it would be important to complement the in vitro studies, by testing the function of Fam81A in synaptic transmission, in vivo (by for example using a virus-mediated knockout/knockdown/labeling approach). We also think that it would be important to address Reviewer 3's comments, and, if possible, to strengthen the meta-analysis performed here, as we think this would increase the value of this as a resource to the community.*

In accordance with Reviewer 1’s suggestion, we have now presented detailed information on the in vivo expression of FAM81A in Figure 3D and 3E.

Regarding the role of FAM81A in synaptic transmission, we would like to respectfully remind the editor that we already showed that the downregulation of FAM81A shRNA significantly reduces the network activity in dissociated neuronal cultures, indicative of reduced synaptic transmission (Fig. 9). Although we could use a similar approach *in vivo* it will be rather incremental and confirmatory. To perform an in-depth analysis of the role of FAM81A in synaptic transmission in vivo, as suggested, would require a very substantial body of further research which is beyond the scope of the present manuscript.

Regarding Reviewer 3's comments, we have strengthened the analysis and enhanced the resource value of our study by the inclusion of new figures (Fig 3F, G) and supplementary tables (Table S3) of proteome data.

**Editor, point 3:**

*As a note Reviewer 2 has raised concerns with the novelty of the study and whether the findings are a good fit for PLOS Biology, given that Fam81A has been observed in the PSD before. After discussion with the Academic Editor, we are less concerned about the conceptual advance of the study if the manuscript is developed further, as outlined above. We do, however, agree with Reviewer 2 that you should not change the name of Fam81A.*

We would like to emphasize that the observation of FAM81A as a PSD component is not a major finding in this paper – the novelty in the paper arises from our characterization of this previously poorly characterized molecule. Indeed, Reviewer 1 states:*“This study provides a nice example of how to identify and understand the roles of previously uncharacterized PSD proteins, expanding our knowledge about the complexity and diversity of the PSD proteome.”*  Reviewer 3 also provides very supportive comments.

We agree to use the name FAM81A and have removed all references to DRACC1 in the manuscript.

In addition to addressing the comments from the editors below, we address the comments from reviewers as much as possible. We addressed 22 of 23 comments raised by the reviewers by additional experiments, analysis, or explanation. The only one comment that we didn’t address was re-analysis of the raw mass spectrometry files of individual dataset (Reviewer 3, point 4). However, as Reviewer 3 indicated, it is beyond the scope of this paper. We sincerely hope the editors find our manuscript now acceptable to PLoS Biology.

# Response to Reviewers

The reviewers’ comments are reproduced in italics with our responses in normal text.

# Reviewer: 1

*Reviewer #1, Kihoon Han (note - Reviewer 1 has signed this review): In this manuscript, Kaizuka et al. used systematic approaches to identify uncharacterized PSD proteins from previous proteomic analyses. Among the 97 candidates, the authors further characterized the top-ranked protein FAM81A, which they renamed as DRACC1. Using biochemical, cell biological, and electrophysiological analyses, the authors provided evidence that DRACC1 can interact with some core PSD proteins and induce LLPS-mediated condensation of the proteins. This study provides a nice example of how to identify and understand the roles of previously uncharacterized PSD proteins, expanding our knowledge about the complexity and diversity of the PSD proteome. Additionally, the manuscript is well-written and describes key findings in a solid manner. I have a few comments that will strengthen the main conclusions of the manuscript:*

We thank the reviewer for their supportive comments. We have addressed their “few comments” below:

**Reviewer 1, point 1:**

*1. Although the authors provide information about the conservation and expression profiles of DRACC1/2 in Figure 2 and Figure 3A, more direct validations of this information at the protein level should be performed.*

We believe the conservation data shown in Figure 2C and 2D does not require any further validation as it is derived directly from Gene2Function database.

To complement and extend the mRNA expression data from different tissues in Figure 2E, we now cite a paper (Dosemeci et al., 2019) that has shown the protein expression profile in 7 tissues (liver, kidney, spleen, heart, brain, muscle, lung) which shows a unique FAM81A expression in the rat brain, consistent with the results shown for human in Figure 2E and mouse in Figure S3A. In addition, we provide protein expression data from across the mouse brain (Figure 3C; low magnification sagittal section of brain stained with antibody to FAM81A) and high-magnification images of FAM81A in many brain regions showing the expression at single-synapse resolution (Figure 3D). In addition, we provide data showing NMDA receptor and FAM81A synaptic localization in the hippocampal formation (Figure 3E).

**Reviewer 1, point 2:**

*Additionally, detailed information about the antibody against DRACC1 (Figure 3A) needs to be included in the manuscript (e.g., specificity between DRACC1 and 2). Once the antibody is validated, brain regional and age-dependent expression, subcellular distribution, etc. can be analyzed with the antibody using at least mouse or rat brain samples.*

We have inserted the following text in the method section:

“ Anti-FAM81A antibody Ab1 is raised for amino acids 86-225 of human FAM81A and Ab2 is raised for amino acids 262-368. Consistent with the previous report (Dosemeci et al., 2019), both antibodies detected a major band above 40 kDa in the mouse brain lysate (Fig. 3A and 3B), the expected size of FAM81A protein, suggesting that the endogenous FAM81A is successfully detected by these antibodies.”

This antibody was used for studies of subcellular distribution (Figure 3A), complex formation (Figure 3B), and tissue expression (Figure 3C-E).

**Reviewer 1, point 3:**

*2. In Figure 4, the authors characterized various deletion mutants of DRACC1 to identify critical regions for the formation of condensates. It is necessary to show whether those different deletion mutants have similar expression levels in cells by western blotting.*

The western blotting data showing the different deletion mutants have similar expression levels is shown in the new Figure S6A-B.

**Reviewer 1, point 4:**

*3. Regarding the formation of condensates by DRACC1, the experimental results suggest that the N-terminal regions are critical while the IDR is in the C-terminus. How can this be explained?*

We have now included the following explanation for this result in the discussion:

“Multivalent protein-protein interactions and intrinsically disordered regions (IDR) are two important factors that drive LLPS (Shin and Brangwynne, 2017). We found that the IDR of FAM81A in the C-terminal half of the protein was not essential for LLPS (Fig. 5). Instead, we found the region between the two coiled-coiled domains (107-157) and the N-terminal domain (1-36) were critical for LLPS, possibly by impairments in their multivalent protein-protein interactions.”

**Reviewer 1, point 5:**

*4. Regarding the protein interaction between DRACC1 and core PSD proteins (Figure 5), the interaction should be validated in vivo using PSD samples.*

As requested, we have now validated the interaction in vivo. See Figure 3B.

**Reviewer 1, point 6:**

*5. In addition, whether such interaction in HEK293T cells requires condensation of DRACC1 can be tested with the deletion constructs used in Figure 4.*

As requested, we performed the interaction studies of the FAM81A mutants with PSD-95 (Fig. 6C) and have discussed the relevance of these results to condensation in the revised discussion.

**Reviewer 1, point 7:**

*6. In Figure 7, the effects of shDracc1 look subtle for both PSD-95 puncta size and spike frequency. Can other parameters, such as excitatory synapse density, dendritic spine size/morphology, etc. be analyzed? In addition, effects of overexpression of WT or deletion mutants of DRACC1 can be tested for the same parameters to further confirm the functional significance of condensation of DRACC1.*

Analysis of the dendritic spine in shRNA induced neurons was not possible due to the low expression level of GFP. Instead, we analyzed the size of dendritic spines on neurons overexpressing FAM81A. As a result, we found that the diameter of dendritic spine in neuron that overexpress full length FAM81A is wider than that of neuron overexpressing ∆107-157 mutant which can’t localize to PSD (Fig. 6B). These data further confirm the role of FAM81A to increase synapse size.

# Reviewer: 2

**Reviewer 2, point 1:**

*Reviewer #2: Here, Kaizuka et al describe the role of the PSD protein FAM81A. The manuscript focus in a meta-analysis of PSD (protein and PPI) datasets to select understudied PSD proteins. Unfortunately, in the way that is presented, the manuscript has very little new information to offer and in the current format is not suitable for publication.*

We respectfully disagree with the assertion that there is “very little new information to offer”. The following is a list of findings that have not previously been reported in the literature to our knowledge:

1. FAM81A forms condensate via liquid-liquid phase separation (LLPS)
2. The region between two coiled-coil domains is important for LLPS of FAM81A
3. LLPS of FAM81A is needed for its PSD localization
4. FAM81A directly interacts with three PSD proteins
5. FAM81A forms condensate together with other PSD proteins
6. FAM81A enlarges the condensate of PSD proteins
7. Loss of FAM81A results in decreased size of PSD
8. Loss of FAM81A results in decreased neuronal activity

In the revised manuscript, we further showed the following findings.

9. PSD localization of FAM81A enlarges dendritic spines

We note that Reviewer 1 is very supportive of our paper and recognizes the novel contributions of our findings.

**Reviewer 2, point 2:**

*It is not clear what the so-called "meta" analysis is, or how it was performed. It seems that is only looking at different datasets and check what proteins are more replicated but with not much information in literature. The authors selected FAM81A as their candidate, perform a series of assays and because of their findings they conclude that the "meta" analysis is a very important tool. However, it seems that this is a hand pick molecule, so I'm not sure what are they validating.*

We have described how the meta-analysis was performed in the Results section “Meta-analysis of PSD proteome datasets”.

In the revised manuscript, we have made sure that we have made no statement that refers to the “meta-analysis is a very important tool”.

**Reviewer 2, point 3:**

*Unfortunately, the candidate selected, FAM81A, has been already characterized as a PSD protein including protein localization by EM. See PMID: 30735723. This makes the whole first part of the manuscript redundant and inaccurate in terms of novelty.*

We respectfully disagree with the statement “*This makes the whole first part of the manuscript redundant and inaccurate in terms of novelty”.* The data presented in the paper from Dosemeci and colleagues (PMID: 30735723) is limited to (i) a western blot showing FAM81A expression in brain, (ii) and in the PSD fraction, and (iii) EM showing that FAM81A is localized in the PSD. I refer the reviewer to our response 1 above that lists 9 areas of novelty in our manuscript.

**Reviewer 2, point 4:**

*The section describing FAM81A PPIs is also redundant as the authors clearly show that PPIs with several PSD proteins including PSD95 and SYNGAP1 have been already described in literature.*

The only information about FAM81A PPI in the literature is that pull down of the PSD proteins recovers FAM81A along with many other proteins (Dosemeci et al. 2007, Fernández et al. 2009, Bayés et al. 2014, Li et al. 2017). This provides no information as the direct or indirect nature of the interaction or the structural features required in FAM81A. Our data have addressed these issues:

1. We validated the interaction of endogenous FAM81A and PSD-95 using immunoprecipitation from brain (Fig. 3B).
2. We showed that cotransfection of FAM81A and PSD-95, GluN2B or SynGAP in HEK cells can be coimmunoprecipited, suggesting that there is no neuronal intermediate protein involved in the interaction (Fig. 7G).
3. We performed a detailed mapping of the required functional domains in FAM81A that are required for the interaction with PSD-95 using deletion mutants, which suggest multiple PSD-95 interacting interfaces in FAM81A (Fig. 6C).

1 and 3 were not included in the original manuscript and are included in this revision.

**Reviewer 2, point 5:**

*The authors then show that FAM81A under certain experimental conditions can induce phase-separation when expressed with their protein interactors. This is expected as most of PPIs can induce phase-separation under the reported experimental conditions. In fact, phase-separation can be viewed as other way to observe PPI. This information is also redundant.*

The reviewer provides no citation or evidence for the claim that “most of PPIs can induce phase-separation under the reported experimental conditions”. While phase-separation require PPIs, it also requires other features such as optimum affinity and most notably, multimeric structure. For example, CaMKII – Ca^2+^/calmodulin interaction does not cause phase separation by itself. It was not known if FAM81A can undergo phase separation until we tested this. We, therefore, strongly disagree with the reviewer’s opinion that the “information is redundant”.

**Reviewer 2, point 6:**

*The authors provide two new pieces of information. One is that the n-terminal region of FAM81A might be necessary for PPI with the reported PSD proteins, and also that knockdown of FAM81A affects PSD size and neuronal activity. While this information is novel I consider it not enough for publication. The manuscript should need to be re-written and focus in these assays and remove what is known, at least not presented as new information.*

We refer to Point 1 above which describes that there are more than 2 novel pieces of information in the manuscript. We have cited known information as appropriate.

**Reviewer 2, point 7:**

SynGO should be used as database analysis instead of GO.

This is an excellent suggestion and we now use SynGO (Fig. 1F, G).

**Reviewer 2, point 8:**

*The authors should not propose to change the protein id of FAM81A for DRACC1. This has no point and only will create problems with future annotations and databases. FAM81A is already reported as a PSD protein, and annotated as this in SynGO. I strongly oppose to a change in the protein id.*

We agree to use the name FAM81A and have removed all reference to DRACC1 in the manuscript.

# Reviewer: 3

*Reviewer #3: The manuscript by Kaizuka et al. describes a meta-analysis of 35 previously published PSD protein datasets, 20 of which are derived from mass spectrometry analysis of enriched PSD fractions and 15 of which are candidate-based using immunoprecipitation, affinity enrichment, or proximity labeling approaches. This meta-analysis could have been done better, nonetheless, it was effective enough to generate interesting candidate PSD proteins for further study. Assuming that proteins common to many datasets are more likely to be bona fide PSD proteins, the authors studied the function of a previously uncharacterized protein, Fam81 (renamed DRACC1) with regards to the PSD. They showed that DRACC1 promotes liquid-liquid phase separation of several major and well-characterized PSD proteins, and down-regulation of DRACC1 in neurons causes a decrease in PSD-95 puncta size and frequency of neuronal firing. Overall the manuscript is well-written, and the approach is a clever example of the utility of proteomic data meta-analysis. Characterization of DRACC1 furthers our understanding of the role of liquid-liquid phase separation in PSD formation and function. The manuscript should be useful for readers who are interested in the fields of PSD composition and function as well as liquid-liquid phase separation.*

We thank this reviewer for reviewing and appreciating our manuscript. We further improved our manuscript based on the comments listed below.

**Reviewer 3, point 1:**

*Specific comments:*

*1. Page 2: Page 4, line 8: Please mention here that the authors have renamed this protein (until I read page 7 it was not clear who had renamed the protein)*

We agree with the suggestion by the editor and Reviewer 2 to use the name FAM81A and have removed all reference to DRACC1 in the manuscript.

**Reviewer 3, point 2:**

*2. last sentence on page 8 (first sentence on page 9): condensates found in both soma and dendritic shafts (Fig 3F, 3G)- it is not clear to me where soma are in Fig 3F and Fig 3G. (figures say PSD and dendritic shafts, respectively, nothing about soma). Do you mean PSD rather than soma? Next sentence, which condensates are moving rapidly compared to those in PSD? Movie S1 shows those in dendritic shaft are moving fast, is that what is meant here? Please clarify.*

We agree with this comment. In the revised manuscript, we showed magnified images of both soma and dendritic shaft (Fig. 4F and 4G). We deleted the description “moving rapidly” and simply explained that no puncta remain same position for 30 min at soma and dendritic shaft as follows.

“In addition to accumulation at dendritic spines, we found the condensates of FAM81A in soma and dendritic shafts (Fig. 4E-4G). These cytoplasmic condensations do not remain in same position for 30 min, in contrast to stable localization of the puncta on PSD (Fig.4E-4G). These results suggests that FAM81A not only accumulates on PSD but also forms dynamic condensates in cytoplasm.”

**Reviewer 3, point 3:**

*3. page 13, lines 3-4: Estimates of the abundance of DRACC1 can be made from the mass spectrometry datasets. One or more high quality MS datasets could be analyzed either by spectral counting (how often are peptides from DRACC1 seen compared to peptides from other proteins, corrected by normalization based on number of theoretical tryptic peptides from DRACC1) or iBAQ (intensity-based quantitation, again normalized to number of theoretical tryptic peptides).*

In the revised manuscript, we evaluated the abundance of FAM81A in PSD and synaptosome using our previous proteome datasets. As a result, we confirmed that FAM81A is enriched in PSD (Fig. 3F and 3G). Quantification with iBAQ showed that FAM81A is a 304th abundant protein out of 3314 total detected protein in the PSD, indicating that FAM81A is indeed one of the major PSD proteins.

**Reviewer 3, point 4:**

*4. While the meta-analysis here was very useful for generating DRACC1 as a very interesting candidate, a better meta-analysis would have included re-analysis of the raw mass spectrometry files when available for each of the MS-based PSD profiles. This is most likely beyond the scope of the current manuscript but would improve the overall meta-analysis, especially since studies have shown that MS data analysis varies significantly between labs.*

We agree that comparison of raw MS data from 35 datasets is indeed “beyond the scope of the current manuscript”.

**Reviewer 3, point 5:**

*5. The attrition rate due to failed ID conversion for proteins was very high in this manuscript. Again, re-analysis of raw MS data would help with this problem, but more attention to renaming the MS datasets for comparison would have been helpful even without direct analysis of MS data. Again, this may be beyond the scope of this manuscript, as the meta-analysis was only a starting point, not the main focus of the paper, and effective enough to ID DRACC1 as an interesting and important PSD component.*

We thank the reviewer for this comment. In the revised manuscript, we described the number of proteins before and after ID conversion (Table 1). The rate of failed ID conversion stands at 0-20.1%, with average 2.3%, a sufficiently low figure that bolsters our arguments. In limited number of cases, the apparent high rate of failed ID conversion is observed because of specific data description. For example, Jordan et al. Mol Cell Proteomics. 2004 3:857-71 reported detection of “452” PSD proteins. This number is much larger than “286”, the number shown in our manuscript. However, they listed ID of only “306” proteins in the paper. Although the reason is unclear, this may be because they counted different isoform as different proteins; e.g. four isoforms of actinin α1.

**Reviewer 3, point 6:**

*Minor comments:*

*1. Page 3, line 5: remove "the" before "detergent"*

We corrected the text.

**Reviewer 3, point 7:**

*2. Other minor grammatical errors remain throughout the manuscript*

In the revised manuscript proofreading by native English speaker is applied.

**Reviewer 3, point 8:**

*3. Figure 2C, "chicken" and "sea squirt" are misspelled*

We corrected the misspellings. Thanks.
