# Supplementary material for: FAM81A is a postsynaptic protein that regulates the condensation of postsynaptic proteins via liquid–liquid phase separation
Source: PLoS Biol. 2024 Mar 7;22(3):e3002006. doi: 10.1371/journal.pbio.3002006 (PMC10919877; doi:10.1371/journal.pbio.3002006)
Supplement: Attachment — Submitted filename: Reply to the editors comments.docx [file pbio.3002006.s017.docx]

**REPLY TO THE EDITOR’S COMMENTS**

1) TITLE: We would like to suggest a minor change to the title to improve its flow. If you agree, we suggest you change it to: "FAM81A is a postsynaptic protein that regulates the condensation of postsynaptic proteins via liquid-liquid phase separation"

We agree with this change.

2) ABSTRACT: Please note that per journal policy, the model system/species studied should be clearly stated in the abstract of your manuscript.

We added this information in the abstract.

3) ETHICS STATEMENT: Please update the ethics statement in your methods section to include the full names of the IACUC/ethics committees that reviewed and approved the animal care and use protocol/permit/project license. Please also include an approval numbers. Please also update this statement to include the specific national or international regulations/guidelines to which your animal care and use protocols adhered. Please note that institutional or accreditation organization guidelines (such as AAALAC) do not meet this requirement.

We updated the ethics statement in the method section, “Mice”. According to the suggestion, we included the full names of the ethics committee, approval numbers, and national guidelines we followed.

4) BLURB: In the relevant section of our online system, please provide a blurb which (if accepted) will be included in our weekly and monthly Electronic Table of Contents, sent out to readers of PLOS Biology, and may be used to promote your article in social media. The blurb should be about 30-40 words long and is subject to editorial changes. It should, without exaggeration, entice people to read your manuscript. It should not be redundant with the title and should not contain acronyms or abbreviations.

We prepared the blurb as follows:

We identified a major- but poorly characterized postsynaptic protein by meta-analysis of proteome datasets. Subsequent analysis revealed that this protein regulates the condensation of postsynaptic proteins via liquid-liquid phase separation to affect the size and function of synapses. (36 words)

5) WESTERN BLOTS: Please note that we require the original, uncropped and minimally adjusted images supporting all blot and gel results reported in an article's figures or Supporting Information files. We will require these files before a manuscript can be accepted so please prepare and upload them now. Please carefully read our guidelines for how to prepare and upload this data: https://journals.plos.org/plosbiology/s/figures#loc-blot-and-gel-reporting-requirements

As instructed, we submit the uncropped and minimally adjusted images of the immunoblotting.

6) CODE: Per journal policy, if any code was generated to support the conclusions of your manuscript, we would require that you make it available without restrictions upon publication. Please ensure that any code is sufficiently well documented and reusable, and that your Data Statement in the Editorial Manager submission system accurately describes where your code can be found.

No specific codes are generated to “support the conclusions of this manuscript”. We just used R codes to make some graphs and are happy to provide those codes upon request.

7) DATA: You may be aware of the PLOS Data Policy, which requires that all data be made available without restriction: http://journals.plos.org/plosbiology/s/data-availability. For more information, please also see this editorial: http://dx.doi.org/10.1371/journal.pbio.1001797

Note that we do not require all raw data. Rather, we ask that all individual quantitative observations that underlie the data summarized in the figures and results of your paper be made available in one of the following forms:

a. Supplementary files (e.g., excel). Please ensure that all data files are uploaded as 'Supporting Information' and are invariably referred to (in the manuscript, figure legends, and the Description field when uploading your files) using the following format verbatim: S1 Data, S2 Data, etc. Multiple panels of a single or even several figures can be included as multiple sheets in one excel file that is saved using exactly the following convention: S1_Data.xlsx (using an underscore).

b. Deposition in a publicly available repository. Please also provide the accession code or a reviewer link so that we may view your data before publication.

Regardless of the method selected, please ensure that you provide the individual numerical values that underlie the summary data displayed in the following figure panels as they are essential for readers to assess your analysis and to reproduce it:

Fig 1D,E; Fig 2A,D,E; Fig 3F-G; FIg 4D; Fig 5C-E; Fig 6B; FIg 8B; Fig 9B,D; Fig S1; Fig S3; Fig S6B; Fig S8

NOTE: the numerical data provided should include all replicates AND the way in which the plotted mean and errors were derived (it should not present only the mean/average values).

Please also ensure that figure legends in your manuscript include information on where the underlying data can be found, and ensure your supplemental data file/s has a legend.

Please ensure that your Data Statement in the submission system accurately describes where your data can be found.

We agree with the policy and share the indicated data as “a. Supplementary files”. We summarized the individual numerical values described in marked panels as “S1 Data” and referred to in the text.

As you address these items, please take this last chance to review your reference list to ensure that it is complete and correct. If you have cited papers that have been retracted, please include the rationale for doing so in the manuscript text, or remove these references and replace them with relevant current references. Any changes to the reference list should be mentioned in the cover letter that accompanies your revised manuscript.

We checked the references. Also, we corrected some mistakes found in the manuscript. We updated the plots shown in Figure 9D and S8A as we noticed the data referred to for these plots were different from the latest version while checking the raw data. This doesn’t affect the conclusion of this study. We apologize for this inconvenience.
